# Understanding the influencing factors of tourists’ revisit intention in traditional villages

**DOI:** 10.1016/j.heliyon.2024.e35029

**Published:** 2024-07-22

**Authors:** Mengyi Lin

**Affiliations:** College of Design and Innovation, Fujian Jiangxia University, Fuzhou, 350108, China

**Keywords:** Traditional villages, Space atmosphere, Information richness, Rural tourism, Revisit intention

## Abstract

The intention of tourists to revisit traditional villages plays a significant role in their sustainable development. This study utilizes a cross-sectional survey design and collects 373 valid responses from Chinese tourists via a questionnaire. The questionnaire, based on a Likert 5-point scale, encompasses key constructs such as space Atmosphere, place attachment, perceived interest, experiential marketing, recreation perception, environmental image perception, well-being, information richness, and revisit intention. To ensure the quality of data, reliability and validity assessments were performed, followed by the verification of research hypotheses using Structural Equation Modeling (SEM). The findings indicate that perceived interest and experiential marketing are pivotal variables influencing tourists' revisit intentions. well-being emerges as a crucial driver that enhancing the likelihood of tourists' return visits. Additionally, recreation perception and perceived interest significantly impact well-being, while information richness positively moderates the effects of space atmosphere, place attachment, perceived interest, and environmental image perception on well-being. These findings can be utilized to formulate strategies that influence tourists' intentions to revisit traditional villages.

## 1 Introduction

Traditional villages are the largest heritage left by Chinese farming civilization [1]. In June 2019, the Ministry of Housing and Urban-Rural Development of China and relevant departments released the fifth batch of traditional Chinese villages. A total of 6819 traditional villages in China are protected. Stimulating tourism in traditional villages is an important means of revitalizing and protecting them [2]. With the intensification of competition among tourism industries, tourists' satisfaction, revisit intention and behavior play an important role in the sustainable development of tourist destinations and scenic spots. Kozak and Rimmington emphasized that tourists’ revisit based on their satisfaction on the destination is extremely important for tourism management and determining the needs of tourists, because revisit is the result of tourists' overall satisfaction in the recreational experience [3]. Oppermann [4] believes that the proportion of revisit tourists to total tourists can be used as an indicator for evaluating the developing potential of tourist destinations and classifying different stages. Therefore, it is crucial to understand the factors that influence tourists' willingness to revisit traditional villages.

Based on past literature on tourism, it is found that tourist perception theories such as destination image perception, place attachment, recreation perception and tourism experience have been used to predict tourists' revisit intention, and their positive impacts have been proved [[5], [6], [7], [8], [9], [10]]. There are also some scholars who have discussed the key factors affecting tourists' decision-making from different perspectives. For example, Zhang's research shows that tourism well-being has a positive impact on their revisit intention [11]. Su’s research verifies that playfulness can improve tourists' intention to revisit [12], and the impact of space atmosphere perception on traveling experience [13]. It can be seen from these studies that the relationship among image perception, place attachment, recreation perception, space atmosphere, traveling experience, playfulness, well-being and revisit intention is crucial for understanding the factors of tourists deciding to revisit destinations. However, few studies have examined such relationship by adding any variables to empirical research or constructing a theoretical framework for all of the above variables, thereby extending the understanding of theoretical development in the new era. This gap in the literature highlights the need for a more comprehensive and integrated approach to understanding the complex interplay between various factors that influence tourists' decisions to revisit traditional villages.

The current research gap is twofold: firstly, there is a lack of a comprehensive theoretical framework that integrates these experiential factors to predict revisit intentions. Secondly, the mediating role of Well-being in the relationship between these factors and revisit intentions has not been thoroughly explored. Addressing these gaps is crucial for developing effective management strategies that cater to the needs and preferences of tourists, thereby fostering the sustainable development of traditional village tourism. This study aims to bridge this gap by identifying and examining the key determinants of tourists' revisit intentions in traditional villages. Specifically, we focus on the role of experiential factors such as Space Atmosphere, Place Attachment, Perceived Interest, Experiential Marketing, Recreation Perception, Environmental Image Perception, and Information Richness, and how these contribute to enhancing Well-being and, consequently, Revisit Intention.By examining the relationships among these variables and their collective impact on Well-being and Revisit Intention, this research seeks to provide a more nuanced and holistic understanding of the factors influencing tourists' revisit behavior, thereby extending the theoretical development in the new era of tourism research.

## 2 Literature review and research hypothesis

### 2.1 Tourism well-being and revisit intention

Traveling and vacationing is an important way to improve well-being [14]. Tourism well-being emphasizes what an individual experience during his or her travel, including the good feeling generated after reaching a certain level of physical fitness, emotion, intelligence and spirit, and the resulting deep cognition [15]. In the context of tourism, the subjective well-being of tourists can contribute to the understanding of tourism impacts by tour operators, policy makers and tourists [16]. Tuo et al. believe that tourism well-being is a certain perception and cognition experienced and generated by tourists, and that tourism well-being of is stronger than general well-being [15]. In the stages before, during and after travel, it can promote the good feeling of tourists by different means such as information stimulation and activity experience. Information stimulation before travel can trigger potential customers to travel to tourist destinations, and the perceived well-being during and after travel will further enhance tourists’ revisit intention and the generation of word-of-mouth.

The research of Uysal et al. shows that tourism experience and activities have a significant impact on the overall life satisfaction of tourists and the well-being of residents [17]. As for the experience cultivation of rural tourism, when the tourist destination meets the recreational needs of urban residents and tourists, people will be integrated into recreational activities and obtain a sense of well-being through such experience which improve recreational benefits [18]. Revisit intention refers to an individual’s intention to revisit the same environment or place as well as recommending the place to others [19], which is also an important indicator of the life cycle and economic benefits of tourist destinations [20]. Kang believes that tourism well-being is the positive emotions generated by tourists in the process of tourism activities and the positive value and significance provided to tourists [21]. Therefore, when tourists are satisfied with the experience at the destination, they may have an intention to revisit the place [10,[22], [23], [24], [25], [26]]. Zhang’s research shows that tourism well-being has a positive effect on tourists’ revisit intention [11]. Therefore, this research proposes the following hypothesis.

H1 There is a significant positive correlation between tourism well-being and tourists’ revisit intention to traditional villages.

### 2.2 Space atmosphere

In many ways, how a space is organized highlights the role that experience might play within it [13]. Kunkel and Berry believes that the colors, smells, sounds, behaviors of servers, temperature and interactions perceived by the senses in the space can all be regarded as space atmosphere [27]. In the past, scholars mostly focused on the research of consumption space such as department store, homestays and restaurants, coffee shops, etc., and the results of the research found that the spatial impression shaped by the space atmosphere will affect consumers’ willingness to consume and their emotional experience [[28], [29], [30], [31], [32]]. Turley and Milliman pointed out that space atmosphere significantly affects people’s perception of the situation, including the interactions among time, task and the predetermined goals, and that it also affects attitudes, staying time and overall satisfaction [33]. Zhang proposed that environmental factors, genetic factors, genetic-environmental factors can affect tourism well-being [34]. De Vaujany et al.'s research argues that tourists can better understand the nature of a destination by considering the space atmosphere of it [13]. In traditional village tourism, space atmosphere refers to the overall impression of the physical and sensory environment perceived by tourists in traditional villages. This concept encompasses the architectural layout of the village, the natural environment, cultural elements, and the sensory experiences of tourists as they interact with the environment. In this study, the assessment of space atmosphere is based on tourists' perceptions of the village's architectural aesthetics, the harmonious integration with the natural landscape, and the cultural characteristics. These factors collectively influence the emotional and psychological state of tourists, thereby affecting their overall satisfaction with the village and their intention to revisit. Based on this conceptualization of space atmosphere, the following hypotheses are proposed to explore its impact on tourism well-being and revisit intention.

H2 Space atmosphere has a significant positive correlation with tourism well-being in terms of visiting traditional villages.

H3 Space atmosphere has a significant positive correlation tourists’ revisit intention to traditional villages.

### 2.3 Place attachment

Place attachment represents the emotional bond that is formed through an individual's experiences, activities, memories, and associated emotions with a specific place, leading to a deep sense of pleasant connection, preference, and ongoing engagement with that special location [35]. Many countries have researched the driving forces of place attachment in the spatial reconstruction of rural settlements. However, there have been few studies relating to place attachment in China [36]. Place attachment originates from people’s various experiences in the specific place and various activities they participate in, which is the attachment of people to the place [37]. Scholars [8,[38], [39], [40]] believe that place attachment is one of the main factors that affect tourists’ well-being positively, and that place attachment has positive effects on tourists’ behavior intention, consumption intention, environmental responsibility, etc. after travel. In regard to revisit intention, scholars in the past have also verified some positive effects of place attachment [9] on revisit intention [41]. In this study, the assessment of place attachment is based on tourists' identification with, emotional engagement with, and willingness to recommend traditional villages to others. This includes visitors' appreciation of the village's culture, history, and natural environment, as well as their interactions and social connections with the local residents. The degree of place attachment reflects the emotional bond tourists have with traditional villages and may influence their overall satisfaction and intention to revisit the village. Therefore, this research proposes the following hypotheses.

H4 Place attachment has a significant positive correlation with tourism well-being in terms of visiting traditional villages.

H5 Place attachment has a significant positive correlation tourists’ revisit intention to traditional villages.

### 2.4 Perceived interest

Unger & Keman defined playfulness as the pleasant mood that people experience when they participate in fascinating activities [42]. Webster et al. characterize playfulness as a motivational characteristic of individuals (trait) or a subjective characteristic of an experience (state) [43], which is formed by personal experience in a specific environment [44]. Travel is also a transaction process of seeking an all-round experience. Chou’s research found that the attractiveness of recreational places and recreational experience can have an intermediary influence on tourists’ revisit intention through customer satisfaction [45]. Su verified that playfulness improves tourists’ revisit intention [12]. Perceived interest, as the user’s mental activity, reflects the interesting, fun and psychologically happy state that the user feels when interacting with the environment [46]. In the field of tourism, it also reflects the inner feelings and happy emotions generated by tourists during sightseeing activities, which can provide tourists with a temporary escape from daily life and make their hearts interesting [[47], [48], [49], [50]]. It can be inferred that travel as a modern way of recreation will also be affected by tourists’ satisfaction of the experience, and the fun and interest perceived in the travel not only bring joy to tourists physically and mentally, but also improves the degree of tourism satisfaction. In this study, the assessment of perceived interest is based on tourists' evaluations of the fun, novelty, and pleasure derived from traditional village tourism activities. This includes their reactions to the entertainment, cultural experiences, and natural environment of the travel destination, as well as how these experiences meet their needs for leisure and entertainment. The degree of perceived interest can reflect tourists' satisfaction with their travel experience and may influence their overall evaluation and intention to revisit the destination.Therefore, this research proposes the following hypotheses.

H6 Perceived interest has a significant positive correlation with tourism well-being in terms of visiting traditional villages.

H7 Perceived interest has a significant positive correlation tourists’ revisit intention to traditional villages.

### 2.5 Experiential marketing

Experiential marketing refers to a marketing strategy that enhances consumers' perception and memory of a brand or product by creating and providing rich sensory and emotional experiences. Schmitt distinguished experiential marketing from traditional marketing, emphasized the multidimensionality of customer experience caused by brand-related stimulus, and proposed five strategic modules: senses, emotions, thinking, actions and the concept of experiential marketing [51]. Research in the past has shown that customers’ evaluation on service and revisit depend on the service experience they have had [52]. In the digital age, it is necessary to implement experiential marketing to build more sustainable customer relationships [53]. Experiential marketing has proven to be an effective strategy that uses multiple models and techniques to create memorable experiences [54]. Through literature analysis, Anne concluded that positive emotions play an important role in stimulating consumers’ desire and consumption decisions during travel [55]. That is to say, the positive emotional experience brought by experiential marketing can affect the behavior of tourists at the destination and ultimately influence their evaluation of the entire travel process.

In this study, the assessment of experiential marketing focuses on tourists' perceptions of the experiences offered in traditional village tourism activities, including their appreciation for the village's design aesthetics, cultural characteristics, interactive activities, and personalized services. Therefore, this research proposes the following hypotheses.

H8 Experiential marketing has a significant positive correlation with tourism well-being in terms of visiting traditional villages.

H9 Experiential marketing has a significant positive correlation tourists’ revisit intention to traditional villages.

### 2.6 Recreation perception

Recreation perception refers to tourists' subjective evaluation and perception of the leisure and entertainment activities and environment provided by the travel destination. In the past, scholars mostly used public spaces such as urban parks as examples to explore the relationship between residents and tourists’ perceived value of urban recreational space and tourism satisfaction and well-being [[56], [57], [58], [59], [60], [61]], which proves that the perceived value of tourists is an important factor affecting their satisfaction and residents’ well-being. Thus, it is inferred that tourists' perception of the destination can have an impact on their satisfaction and well-being. Many scholars have confirmed that there is a connection between the revisit behavior or revisit intention and the satisfaction of the recreational experience [[62], [63], [64], [65], [66]]. Recreational experience is an experience of intrinsic reward, and the main driving force that makes people to engage in recreational activities is the “pleasant feeling” brought about by this intrinsic reward [67]. Beeho and Ross believe that if tourists are satisfied with their recreational experience, they will recommend the destination to friends and relatives around [68]. In this study, the assessment of recreation perception focuses on how tourists evaluate the performance of traditional village tourism activities in meeting their leisure and entertainment needs. It involves evaluating the destination's performance and quality in satisfying their needs for relaxation, leisure, and entertainment. Therefore, this research proposes the following hypotheses.

H10 Recreation perception has a significant positive correlation with tourism well-being in terms of visiting traditional villages.

H11 Recreation perception has a significant positive correlation tourists’ revisit intention to traditional villages.

### 2.7 Environmental image perception

Environmental Image Perception refers to tourists' overall impressions and evaluations of the environment at a travel destination, including their cognitive and emotional responses to the natural environment, cultural background, social atmosphere, and the destination's sustainable development practices. The tourism industry, having evolved through stages of being resource-driven, product-driven, market-driven, and marketing-driven, has now entered an image-driven era [69]. A friendly environment is not only an important foundation for the sustainable development of tourism, but also a unique attraction for tourists, so it is necessary for tourist destinations to maintain or improve the quality of their environment [70].

Su et al. divided the environmental attributes into natural environment, artificial facilities and cultural environment, investigated the relationship between satisfaction and environmental attributes, place attachment and revisit intention, and confirmed that environmental image perception can have a positive effect on tourists’ revisit intention [19]. Chen’s research shows that physical facilities, environmental image perception and information and facility convenience have a significant positive impact on tourist satisfaction, and tourism image perception has a significant positive impact on tourists’ revisit intention [71]. Therefore, a good environmental image of the destination can effectively increase the revisit rate of tourists [[72], [73], [74]]. Yuan et al. believe that tourists’ perception of the environmental image of traditional villages can reflect their overall impression and evaluation of traditional villages, so it can be used as an important basis for judging whether the local culture has become the value center of tourists’ perception or the main attraction [75]. As a result, a good environment for tourist experience and rich value perception will have a positive effect on cultivating tourists’ loyalty to ancient villages, thereby increasing their revisit intention [76]. In this study, the assessment of environmental image perception focuses on how tourists evaluate the natural environment of traditional villages, the preservation of cultural heritage, and the villages' attractiveness and friendliness to visitors. This involves evaluations of the cleanliness, completeness of facilities, reasonableness of consumption, traffic planning, and overall comfort of the villages. Therefore, this research proposes the following hypotheses.

H12 Environmental image perception has a significant positive correlation with tourism well-being in terms of visiting traditional villages.

H13 Environmental image perception has a significant positive correlation tourists’ revisit intention to traditional villages.

### 2.8 Information richness

Daft and Lengel first put forward the concept of information richness [77], which is an important factor in the choice of media and affects consumers’ cognitive ability of products and channels. There are varying degrees of information richness among communication technology tools [78]. With the advancement of Information and Communication Technology (ICT), tourism-related applications are continuously being improved to respond to tourists’ preferences and needs [79]. As a result, theoretical research on media richness has become a hot spot, and it has been widely introduced into the research on Internet activities [[80], [81], [82]]. However, few scholars analyze the influence of information richness on tourism behavior. What needs to be distinguished is that media richness theory mainly confirms the importance of communication factors, such as interactivity and vividness, in facilitating communication [83]. Information richness refers to the amount of information that can be conveyed through the media [84,85]. Patrakosol & Lee believe that information richness allows individuals to clearly understand necessary information and appropriately acts as a bridge to communicate with consumers [86], stimulating consumers' potential needs by displaying detailed product information to them. When the information content is rich, consumers' confidence in the brand and product will increase [87].

Based on this concept, we define the perception of information richness as the abundance of information or product-related knowledge provided to tourists at the travel destination. The assessment of information richness focuses on how tourists evaluate the quality and quantity of information offered during traditional village tourism activities. This includes evaluations of cultural background information, activity details, service introductions, and other relevant information that tourists might be interested in. Therefore, this research proposes the following hypotheses.

H14 Information richness plays a significant positive moderating role in the influence path of space atmosphere on tourism well-being.

H15 Information richness plays a significant positive moderating role in the influence path of place attachment on tourism well-being.

H16 Information richness plays a significant positive moderating role in the influence path of perceived interest on tourism well-being.

H17 Information richness plays a significant positive moderating role in the influence path of experiential marketing on tourism well-being.

H18 Information richness plays a significant positive moderating role in the influence path of recreation perception on tourism well-being.

H19 Information richness plays a significant positive moderating role in the influence path of environmental image perception on tourism well-being.

**Fig. 1:**
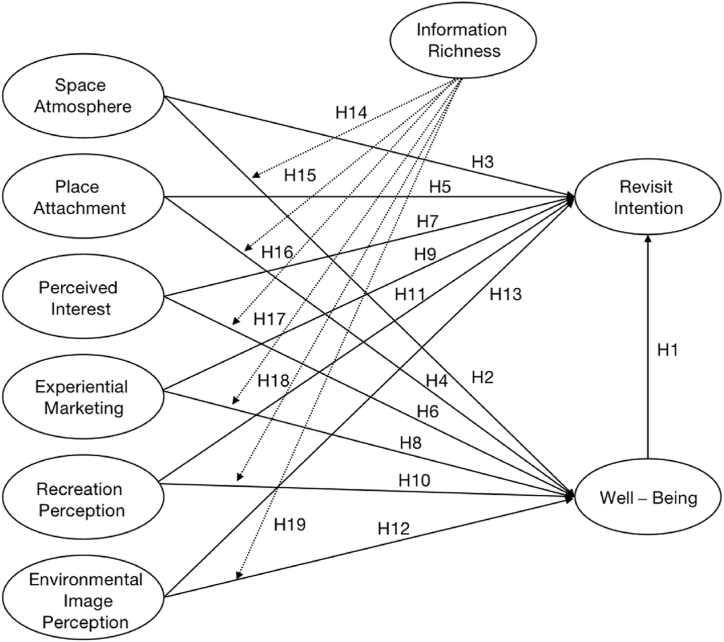
Concept model.

Therefore, based on the above analysis and hypotheses, the theoretical model of this research is constructed (see Fig. 1).

## 3 Research design and methods

### 3.1 Questionnaire design

This study adopts a quantitative research design to investigate the influencing factors of tourists' revisit intention in traditional villages. The research model is developed based on a comprehensive literature review and theoretical framework construction. The primary research method employed in this study is a survey method, utilizing a self-administered questionnaire to collect data from tourists who have visited traditional villages.

The questionnaire items and reference sources are shown in Table 1. To measure SA, four items of Baker [88] and Turley & Milliman [33] were adopted, such as "The architectural layout of this traditional village is reasonable". The PA measurement is borrowed from Tuan [35] and Meng [40], such as "This traditional village is the best tourist destination for me to quickly experience rural folk culture". The PI assessment is based on three items from Moon & Kim [40]. Schmitt's [51] EM measurement scale, RP of Kozak & Rimmington [3] and Petrick [66]. EIP of Su et al., [19]. WB refers to Keng [21] and Uysal et al. [17], such as“Overall, I am very satisfied with this tour to traditional village.” The 4 items about IR is adopted from Chi & Qu [73] and Purdy et al. [85], such as “In addition to relaxing my mind, this traditional village also provides me a lot of rural folk culture knowledge". Finally, we used Zhang’s [11] 4 items to measure RI, such as "I’m thinking about revisiting this traditional village next time". As shown in Table 1.

**Table 1:** Measurement scale.

Latent Variable	Coding	Item	Source
Space atmosphere	SA1	The architectural layout of this traditional village is reasonable.	(Baker et al., 1992; Turley & Milliman, 2000)
	SA2	The traditional village has a complete range of entertainment facilities.	
	SA3	The staff in this traditional village treat people with enthusiasm and attentive service.	
	SA4	The cultural architecture of this traditional village blends perfectly with the natural scenery.	
Place attachment	PA1	I am most satisfied with the folk culture in this traditional village.	(Meng, 2019; Tuan, 1977)
	PA2	The traditional village gives me the feeling that other places cannot give it.	
	PA3	This traditional village is the best place for me to experience rural folk culture in a short time.	
	PA4	I very much agree with the rural folk culture displayed by this traditional village.	
	PA5	For me, this traditional village has a special meaning.	
	PA6	I admire the environment of this traditional village very much.	
Perceived interest playfulness	PI1	For me, visiting this traditional village is very interesting.	Moon & Kim, 2001)
	PI2	For me, visiting this traditional village is very novelty.	
	PI3	During visiting this traditional village, it makes me feel happy to share my travel experience.	
Experiential marketing	EM1	The design style of this traditional village is in line with my aesthetics.	(Schmitt, 1999)
	EM2	The characteristic products displayed in this traditional village are attractive to me.	
	EM3	The folk activities in this traditional village are familiar to me.	
	EM4	Being in this traditional village can remind me of the past.	
Recreation perception	RP1	This traditional village can meet my needs for relaxation and entertainment.	(Kozak & Rimmington, 2000; Petrick, 1999)
	RP2	During visiting this traditional village, I managed to adjust my emotions.	
	RP3	This traditional village can achieve my purpose of recreation.	
Environmental image perception	EIP1	The overall environment of this traditional village is comfortable and tidy.	(Su et al., 2018)
	EIP2	The traditional village has fully equipped dining and entertainment facilities.	
	EIP3	All the consumption and commodity prices in this traditional village are reasonable.	
	EIP4	The traffic volume in this traditional village is suitable.	
	EIP5	The traffic planning in this traditional village is reasonable and parking is convenient.	
Well-being	WB1	Overall, I am very satisfied with this traditional village tour.	(Keng, 2011; Uysal et al., 2016)
	WB2	I think the journey is full of fun.	
	WB3	I have been very enthusiastic during this trip.	
	WB4	I feel joy and excitement during this journey.	
Information richness	IR1	In addition to relaxing my mind, this traditional village also displayed ancient rural folk culture costumes and architecture.	(Chi & Qu, 2008; Purdy et al., 2000)
	IR2	In addition to relaxing my mind, this traditional village can also tell me a lot of knowledge about rural folk culture.	
	IR3	In addition to relaxing my mind, this traditional village also conveys correct traditional cultural values.	
	IR4	When traveling in this traditional village, I learned the meaning of many traditional cultural symbols.	
Revisit intention	RI1	I am going to visit this traditional village again next time.	(Zhang, 2018)
	RI2	I may come to this traditional village again in the future.	
	RI3	This traditional village will be my first choice for experiencing rural folk culture in the future.	
	RI4	I will introduce relatives and friends to visit this traditional village.	

### 3.2 Data collection and analysis

This research utilizes a questionnaire survey to explore the relationships among the study's key dimensions. The sampling technique employed is convenience sampling, focusing on tourists visiting traditional villages in China. The data collection instrument is a structured questionnaire, divided into two parts: demographic characteristics (Part 1) and behavioral measures related to the research model (Part 2). Part 1 gathers respondents' demographic information, including gender, age, education level, marital status, monthly income, and other basic personal details. Part 2 consists of 37 items corresponding to the research model's constructs: space atmosphere, place attachment, perceived interest, experiential marketing, recreation perception, environmental image perception, well-being, information richness, and revisit intention. These items are based on established scales from prior research and adapted for the context of traditional village tourism, utilizing a Likert Scale ranging from "strongly disagree" to "strongly agree." To ensure the reliability, rationality, and validity of the research, a pre-test was conducted on 100 respondents before distributing the questionnaires. The number, order, and content of the questions were revised based on their feedback, and the final version was improved and distributed on a large scale.

The questionnaire was distributed online through a Chinese questionnaire collection website from July to August 2021. For validity, questionnaires were selected based on three criteria: completion of all questions, diversity in responses, and prior visitation to traditional villages. A priori power analysis was conducted using G-Power to determine the required sample size for the study. Considering a significance level (α) of 0.05, a desired power (1 - β) of 0.95, an effect size (f^2) of 0.15, and the number of predictors in the model being 14, the analysis indicated that a minimum of 194 participants would be required. To account for potential data loss or incompletion, the sample size was increased by 20 %, resulting in a target sample size of at least 243 participants.

After excluding any invalid or incomplete questionnaires, a total of 373 valid questionnaires were collected, yielding a response rate of 94 %. This sample size is more than sufficient for Structural Equation Modeling (SEM), which requires a ratio of at least 10 times the number of analysis items.

Data analysis was conducted using SPSS 26.0 for descriptive statistical analysis to comprehend the overall information derived from the responses. The demographic profile of the respondents is as follows: predominantly aged 26–35 (62.47 %), with a near-even gender split (50.13 % female, 49.87 % male), and mostly employed as company staff and civil servants. The demographic information is detailed in Table 2.

**Table 2:** Demographic information (N = 373).

Frequency Analysis Results			
Items	Categories	Frequency	Percent (%)
Gender	Male	186	49.87
	Female	187	50.13
Age	18–25	94	25.2
	26–35	233	62.47
	36–45	30	8.04
	Over 46	16	4.29
Education level	Junior high school and below	78	20.91
	Technical secondary school and high school	88	23.59
	College and undergraduate	179	47.99
	Master degree and above	28	7.51
Profession	Student	46	12.33
	Civil servant and employee of public institutions	86	23.06
	Company employee	140	37.53
	Self-employed	52	13.94
	Professional technical staff skill	34	9.12
	Retired	5	1.34
	Other	10	2.68
Marriage	Married	270	72.39
	Unmarried	103	27.61
Salary	Below 3000RMB	76	20.38
	3000-6000RMB	118	31.64
	6000-10000RMB	110	29.49
	10000-15000RMB	47	12.6
	Over 15000RMB	22	5.9
Total		373	100

Subsequently, Structural Equation Modeling (SEM) was utilized to test the research hypotheses and evaluate the relationships between variables. Employing AMOS software version 23, SEM was selected for its capacity to examine measurement invariance and estimate both direct and indirect effects within our hypothesized model. The model fit was rigorously assessed using standard indices, such as Comparative Fit Index (CFI) and Root Mean Square Error of Approximation (RMSEA), ensuring the analytical approach was adequate. SEM's robustness in managing large datasets and its prevalent application in social sciences made it the most suitable method for our research.

## 4 Results

### 4.1 Reliability and validity

#### 4.1.1 Confirmatory factor analysis

This research adopts AMOS 23.0 for confirmatory factor analysis (CFA), and uses combined reliability (CR) and average variance extraction (AVE) to analyze the convergent validity of the scale. In the hypothetical model of this research, there are 9 latent variables, namely, space atmosphere, place attachment, perceived interest, experiential marketing, recreation perception, environmental image perception, well-being, information richness and revisit intention.

According to the criterion of model fitting index [89,90], the model fitting indexes of the confirmatory factor analysis model in this research all meet the standard (X2/df = 1.161 < 3, NFI = 0.923 > 0.9, IFI = 0.989 > 0.9, TLI = 0.987 > 0.9, CFI = 0.988 > 0.9, GFI = 0.911 > 0.8, RMSEA = 0.021 < 0.08).

#### 4.1.2 Convergent validity

It can be seen from Table 3 that the standardized factor loadings of the 37 measurement indicators in the model range from 0.682 to 0.882, all greater than 0.50, and the corresponding significance P values are all less than 0.05, indicating that the latent variables and the observed variables have significant mutual influence [91], and each latent variable corresponding to the question is highly representative. Meanwhile, the AVE value of each latent variable is greater than 0.5, and the CR value is greater than 0.7, indicating that the convergent validity of the scale is relatively ideal.

**Table 3:** The results of convergent validity.

			Standardized Factor Loading	Estimated	S.E.	C.R.	P	AVE	CR
SA1	<---	Space atmosphere	0.724	1				0.531	0.819
SA2	<---	Space atmosphere	0.730	0.973	0.078	12.439	a		
SA3	<---	Space atmosphere	0.745	0.990	0.078	12.653	a		
SA4	<---	Space atmosphere	0.716	0.972	0.079	12.232	a		
PA1	<---	Place attachment	0.858	1				0.707	0.935
PA2	<---	Place attachment	0.818	0.900	0.045	19.909	a		
PA3	<---	Place attachment	0.841	0.977	0.047	20.898	a		
PA4	<---	Place attachment	0.830	0.933	0.046	20.396	a		
PA5	<---	Place attachment	0.857	0.988	0.046	21.607	a		
PA6	<---	Place attachment	0.840	0.976	0.047	20.840	a		
PI1	<---	Perceived interest	0.783	1				0.624	0.832
PI2	<---	Perceived interest	0.820	1.080	0.073	14.827	a		
PI3	<---	Perceived interest	0.765	0.948	0.067	14.154	a		
EM1	<---	Experiential marketing	0.818	1				0.649	0.881
EM2	<---	Experiential marketing	0.800	1.003	0.059	16.924	a		
EM3	<---	Experiential marketing	0.807	0.963	0.056	17.114	a		
EM4	<---	Experiential marketing	0.798	1.012	0.060	16.872	a		
RP1	<---	Recreation perception	0.728	1				0.566	0.796
RP2	<---	Recreation perception	0.798	1.094	0.086	12.722	a		
RP3	<---	Recreation perception	0.728	0.966	0.080	12.111	a		
EIP1	<---	Environmental image perception	0.767	1				0.525	0.846
EIP2	<---	Environmental image perception	0.755	0.966	0.069	14.072	a		
EIP3	<---	Environmental image perception	0.712	0.921	0.070	13.247	a		
EIP4	<---	Environmental image perception	0.703	0.932	0.071	13.076	a		
EIP5	<---	Environmental image perception	0.682	0.897	0.071	12.660	a		
WB1	<---	Well-being	0.848	1				0.688	0.898
WB2	<---	Well-being	0.855	1.007	0.05	20.334	a		
WB3	<---	Well-being	0.858	1.060	0.052	20.463	a		
WB4	<---	Well-being	0.751	0.884	0.053	16.724	a		
IR1	<---	Information richness	0.828	1				0.656	0.884
IR2	<---	Information richness	0.808	0.976	0.057	17.242	a		
IR3	<---	Information richness	0.785	0.927	0.056	16.623	a		
IR4	<---	Information richness	0.817	0.965	0.055	17.477	a		
RI1	<---	Revisit intention	0.874	1				0.755	0.925
RI2	<---	Revisit intention	0.856	0.979	0.044	22.168	a		
RI3	<---	Revisit intention	0.863	1.004	0.045	22.512	a		
RI4	<---	Revisit intention	0.882	1.062	0.045	23.437	a		

Note: *p < 0.05, **p < 0.01.

a p < 0.001.

### 4.2 Structural equation modeling

**Fig. 2:**
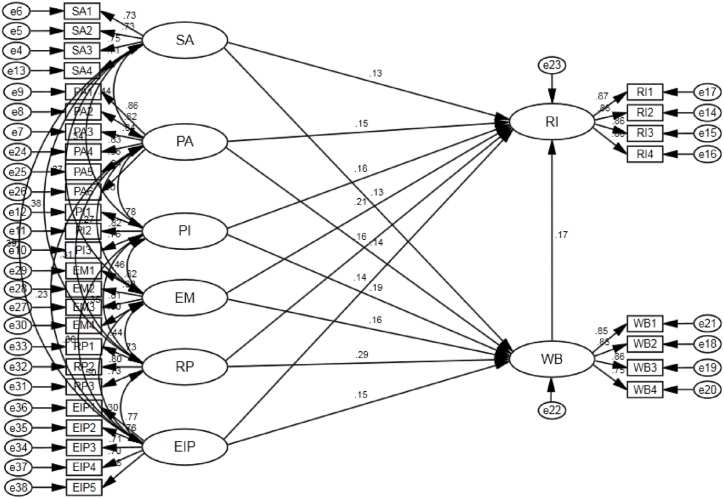
SEM of consumer experience factors in the field of traditional village tourism drawn by AMOS.

According to the conclusions above, the structural equation modeling of consumer experience factors in traditional villages has a total of 8 latent variables, namely: space atmosphere, place attachment, perceived interest, experiential marketing, recreation perception, environmental image perception, well-being and revisit intention, corresponding to which there are 33 observed variables. The results of the structural equation modeling of consumer experience factors in traditional villages drawn by AMOS23 are shown in Fig. 2 below.

According to the evaluation criteria of model fitting indicators, the values of each SEM model fitting indicator in this research meet the research standard (X2/df = 1.168 < 3, NFI = 0.931 > 0.9, IFI = 0.989 > 0.9, TLI = 0.988 > 0.9, CFI = 0.989 > 0.9, GFI = 0.920 > 0.8, RMSEA = 0.021 < 0.08). Therefore, the structural equation modeling has a good fitting effect on the sample data obtained by the questionnaire.

#### 4.2.1 Results of path coefficient test

The results of the path coefficient test in Table 4 show that space atmosphere, place attachment, perceived interest, experiential marketing, recreation perception and environmental image perception have a significant positive impact on well-being (β = 0.133, p < 0.05), (β = 0.137, p < 0.05), (β = 0.187, p < 0.05), (β = 0.162, p < 0.05), (β = 0.289, p < 0.05), (β = 0.152, p < 0.05), so the hypotheses hold. Space atmosphere, place attachment, perceived interest, experiential marketing, recreation perception, environmental image perception and well-being have a significant positive effect on revisit intention (β = 0.130, p < 0.05), (β = 0.147, p < 0.05), (β = 0.183, p < 0.05), (β = 0.208, p < 0.05), (β = 0.159, p < 0.05), (β = 0.141, p < 0.05), (β = 0.165, p < 0.05), and the hypotheses hold.

**Table 4:** The results of path coefficient test.

			Standardized Path Coefficient	Estimated	S.E.	C.R.	P	Test Results
Well-being	<---	Space atmosphere	0.133	0.146	0.063	2.293	0.022	valid
Well-being	<---	Place attachment	0.137	0.127	0.046	2.776	0.006	valid
Well-being	<---	Perceived interest	0.187	0.188	0.055	3.391	a	valid
Well-being	<---	Experiential marketing	0.162	0.169	0.062	2.702	0.007	valid
Well-being	<---	Recreation perception	0.289	0.329	0.066	4.949	a	valid
Well-being	<---	Environmental image perception	0.152	0.165	0.061	2.696	0.007	valid
Revisit intention	<---	Space atmosphere	0.130	0.145	0.058	2.485	0.013	valid
Revisit intention	<---	Place attachment	0.147	0.140	0.042	3.317	a	valid
Revisit intention	<---	Perceived interest	0.183	0.187	0.052	3.618	a	valid
Revisit intention	<---	Experiential marketing	0.208	0.220	0.058	3.829	a	valid
Revisit intention	<---	Recreation perception	0.159	0.185	0.063	2.931	0.003	valid
Revisit intention	<---	Environmental image perception	0.141	0.157	0.056	2.773	0.006	valid
Revisit intention	<---	Well-being	0.165	0.169	0.062	2.710	0.007	valid

Note: *p < 0.05, **p < 0.01.

a p < 0.001.

### 4.3 Results of mediation effect

This research adopts the Bootstrap method in Amos23 to test the mediation effect. The sample size was set as 5000 (usually 1000 or above), and the confidence level of the interval was set as 95 % (usually set as 90 %, 95 % or 99 %). The upper and lower limits were observed based on bias-corrected confidence intervals. If the bias-corrected confidence interval of indirect effect does not include 0, it indicates that the mediation effect exists.

The results of the mediation effect test (Table 5) show that in the model with environmental image perception, recreation perception, experiential marketing, perceived interest, place attachment and space atmosphere as the independent variable, revisit intention as the dependent variable, and well-being as the mediating variable, the values of the indirect effect are 0.025, 0.048, 0.027, 0.031, 0.023 and 0.022, and the confidence intervals of Bootstrap are [0.004, 0.067][0.011, 0.103][0.003, 0.071][0.006, 0.076][0.003, 0.059][0.002, 0.065], in which 0 is not included, indicating that well-being has a significant mediation effect on the impact of environmental image perception, recreation perception, experiential marketing, perceived interest, place attachment and space atmosphere on revisit intention.

**Table 5:** The results of mediation effect.

Mediation Model	Effect	BootSE	BootLLCI	BootULCI
Total Effect				
environmental image perception-->well-being-->revisit intention	0.166	0.051	0.068	0.271
recreation perception-->well-being-->revisit intention	0.207	0.053	0.107	0.313
experiential marketing-->well-being-->revisit intention	0.234	0.057	0.124	0.349
perceived interest-->well-being-->revisit intention	0.214	0.054	0.111	0.322
place attachment-->well-being-->revisit intention	0.170	0.045	0.082	0.260
space atmosphere-->well-being-->revisit intention	0.152	0.052	0.054	0.258
Direct Effect				
environmental image perception-->well-being-->revisit intention	0.141	0.052	0.042	0.246
recreation perception-->well-being-->revisit intention	0.159	0.055	0.054	0.273
experiential marketing-->well-being-->revisit intention	0.208	0.058	0.096	0.322
perceived interest-->well-being-->revisit intention	0.183	0.055	0.080	0.296
place attachment-->well-being-->revisit intention	0.147	0.046	0.060	0.241
space atmosphere-->well-being-->revisit intention	0.130	0.053	0.032	0.237
Indirect Effect				
environmental image perception-->well-being-->revisit intention	0.025	0.015	0.004	0.067
recreation perception-->well-being-->revisit intention	0.048	0.024	0.011	0.103
experiential marketing-->well-being-->revisit intention	0.027	0.017	0.003	0.071
perceived interest-->well-being-->revisit intention	0.031	0.017	0.006	0.076
place attachment-->well-being-->revisit intention	0.023	0.014	0.003	0.059
space atmosphere-->well-being-->revisit intention	0.022	0.015	0.002	0.065

### 4.4 Results of moderating effect test

This research adopts PROCESS analysis in SPSS and tries to verify the hypotheses of moderating effect in this research through Model 1. The results are as follows.

It can be seen from Table 6 that the interactive terms of space atmosphere, place attachment, perceived interest, environmental image perception and information richness show significance (B = 0.216, p < 0.05), (B = 0.125, p < 0.05), (B = 0.133, p < 0.05), (B = 0.096, p < 0.05), which means that information richness has a significant positive moderating effect on the influence paths of space atmosphere, place attachment, perceived interest and environmental image perception on well-being, while the interactive terms of experiential marketing, recreation perception and information richness show no significance (B = 0.079, p > 0.05), (B = 0.021, p > 0.05), which means that information richness has no significant positive moderating effect on the influence path of experiential marketing and recreation perception on well-being.

**Table 6:** Moderating effects of information richness.

variable		dependent variable: well-being					
		Model1	Model2	Model3	Model4	Model5	Model6
independent variable	constant	3.198^c^	3.228^c^	3.230^c^	3.242^c^	3.246^c^	3.239^c^
	space atmosphere	0.389^c^					
	place attachment		0.321^c^				
	perceived interest			0.372^c^			
	experiential marketing				0.453^c^		
	recreation perception					0.452^c^	
	environmental image perception						0.429^c^
moderator	information richness	0.335^c^	0.331^c^	0.308^c^	0.314^c^	0.273^c^	0.317^c^
interactive term	space atmosphere^a^information richness	0.216^c^					
	place attachment^a^information richness		0.125^b^				
	perceived interest^a^information richness			0.113^b^			
	experiential marketing^a^information richness				0.079		
	recreation perception^a^information richness					0.021	
	environmental image^a^information richness perception						0.096^a^
R2		0.285	0.244	0.282	0.316	0.294	0.257
F		48.996^c^	39.800^c^	48.334^c^	56.899^b^	51.218^b^	43.798^c^

a p < 0.05.

b p < 0.01.

c p < 0.001.

Through the simple slope test, the moderating effect of information richness on the influence paths of space atmosphere, place attachment, perceived interest and environmental image perception on well-being are verified respectively. The results are listed as below (Table 7).

**Table 7:** Conditional effects of the focal predictor at values of the moderator.

Conditional effects of the focal predictor at values of the moderator							
moderating path	Moderator variable	B	SE	t	p	95 % CI	
space atmosphere-->well-being	Average value	0.389	0.047	8.226	0.000	0.296	0.481
	High level (+1SD)	0.618	0.067	9.264	0.000	0.488	0.749
	Low level (-1SD)	0.159	0.064	2.486	0.013	0.034	0.285
place attachment-->well-being	Average value	0.321	0.045	7.164	0.000	0.233	0.409
	High level (+1SD)	0.455	0.061	7.480	0.000	0.336	0.574
	Low level (-1SD)	0.188	0.069	2.718	0.007	0.052	0.324
perceived interest-->well-being	Average value	0.372	0.042	8.843	0.000	0.290	0.454
	High level (+1SD)	0.492	0.058	8.435	0.000	0.378	0.607
	Low level (-1SD)	0.252	0.061	4.159	0.000	0.133	0.371
environmental image perception-->well-being	Average value	0.429	0.050	8.591	0.000	0.331	0.527
	High level (+1SD)	0.531	0.071	7.505	0.000	0.393	0.670
	Low level (-1SD)	0.327	0.066	4.969	0.000	0.198	0.456

The results show that when information richness is at a low level, the effect of space atmosphere, place attachment, perceived interest and environmental image perception on well-being is weak (B = 0.159, 95%CI = [0.034, 0.285])(B = 0.188, 95%CI = [0.052, 0.324])(B = 0.252, 95%CI = [0.133, 0.371])(B = 0.327, 95%CI = [0.198, 0.456]), 0 excluded.

When information richness is at a medium level, the effect of space atmosphere, place attachment, perceived interest and environmental image perception on well-being is enhanced (B = 0.389, 95%CI = [0.296, 0.481])(B = 0.321, 95%CI = [0.233, 0.409])(B = 0.372, 95%CI = [0.290, 0.454])(B = 0.429, 95%CI = [0.331, 0.527]), 0 excluded.

When information richness is at a high level, the effect of space atmosphere, place attachment, perceived interest and environmental image perception on well-being is the greatest (B = 0.618, 95 % CI = [0.488, 0.749])(B = 0.455, 95%CI = [0.336, 0.574])(B = 0.492, 95%CI = [0.378, 0.607])(B = 0.531, 95%CI = [0.393, 0.670]), 0 excluded.

## 5 Discussion

The findings of this study offer valuable insights into the determinants of tourists' revisit intentions in traditional villages. Hypotheses H1 through H13, designed to explore the relationships between various experiential factors and tourists' well-being and revisit intentions, were supported by the data. This support indicates a significant positive correlation between the examined constructs and tourists' well-being, which subsequently influences their intention to revisit.

The positive impact of place attachment (H4 and H5) and perceived interest (H6 and H7) on well-being and revisit intentions corroborates previous research that underscores the significance of emotional connections and engaging experiences in fostering loyalty and return behavior [12,38,41,45]. Furthermore, the notable role of experiential marketing (H8 and H9) in enhancing tourists' well-being aligns with studies highlighting the effectiveness of immersive marketing strategies in crafting memorable consumer experiences [55].

However, our findings diverge from existing literature in the context of environmental image perception (H12 and H13). The effect's magnitude appears more pronounced in traditional villages, suggesting that the unique cultural and environmental aspects may exert a stronger influence on tourists' perceptions and behaviors than previously estimated [75]. This divergence could be attributed to the distinctive appeal of traditional villages, rooted in their cultural heritage and authenticity, which might amplify the impact of environmental cues on tourists' evaluations. Additionally, the experiential nature of traditional village tourism, often involving direct interactions with local culture and communities, could foster deeper emotional connections and heightened well-being among visitors.

The mediating effect of well-being between the independent variables—space atmosphere, place attachment, perceived interest, experiential marketing, recreation perception, and environmental image perception—and the dependent variable—revisit intention—was significant. This finding underscores the centrality of well-being in shaping tourists' decision-making processes. It implies that enhancing tourists' well-being through various experiential factors can effectively encourage repeat visits to traditional villages.

The results of the moderating effect test of information richness indicate a significant positive moderating effect on the influence paths of space atmosphere, place attachment, perceived interest, and environmental image perception on well-being. This suggests that the folk architecture, folk costumes, and local customs in traditional villages can improve tourists' overall environmental evaluation and adjust to local life and entertainment more quickly. Such unique experiences, distinct from daily life, are full of freshness and have positive significance and effects on tourists' revisit intentions and exploration of similar traditional villages in the future.

The non-significance of H17 and H18 warrants consideration. While experiential marketing has been proven effective, information richness did not significantly moderate the influence path of experiential marketing and recreation perception on well-being. This finding suggests that while experiential marketing is a valuable strategy, the role of information richness in this context may be less pronounced. Therefore, future differentiated marketing designs for traditional villages could benefit from enhanced exposure and display of folk culture during experiential marketing and recreation perception to improve its impact.

## 6 Conclusion and future research

### 6.1 Conclusion

The contribution of this study is to provide new insights for tourism marketing strategies and consumer behavior research. This study is the first to connect tourists’ perception of the destination’s space atmosphere, place attachment, perceived fun, experiential marketing, recreation perception, environmental image perception and tourists' well-being and revisit intention, constructing a influence model of tourists' revisit intention to traditional villages.

Firstly, although previous literature in tourism has explored the factors affecting tourists’ revisit intention through tourist perception theories such as destination image perception, place attachment, recreation perception and tourism experience [[5], [6], [7], [8], [9], [10],^22^,^26^], perceived interest, space atmosphere and tourism well-being are rarely included in revisit intention model. The empirical results of this research confirm the positive effects of recreation perception, experiential marketing and perceived interest, as well as the significant effect of space atmosphere on tourism well-being.

Managers and developers of traditional village tourism should focus on the deployment of daily work in the above aspects, especially the factors positively correlated to the improvement of tourists’ well-being and revisit intention. A more sustainable way of content output through space atmosphere, place attachment, perceived interest, experiential marketing, recreation perception and environmental image perception should be adopted. Meanwhile, add immersive experience such as social media, online travel programs, and VR to traditional village tourism will enhance the attractiveness of traditional villages to tourists through promoting strengths and avoiding weaknesses on the influence path of information richness on related results, through VR, and ultimately trigger tourists’ desire to revisit, forming an operation method with tourists as the core and establishing differentiated advantages of traditional villages in market competition, further promoting the establishment and operation of sustainable development paths.

Last but not least, the research results can provide effective marketing strategies and guidelines for the development and operation of traditional village tourism.

1. Combined with the analysis model established by this study, formulate a marketing strategy centered on improving tourists' revisit intention to improve tourists' satisfaction by adjusting the factors that affect tourists' revisit intention, and achieve the benign operation of tourism projects and sustainable development of tourism destinations:
1) Improving village environment, adding interactive facilities, developing scientific tour route in traditional villages and ancient dwellings, and improving the service quality and enriching the content of travel can provide tourists a place to relax and improve their well-being of the space atmosphere in traditional villages, so as to influence tourists’ decision to revisit the destination.
2) In the process of traditional village touring, guide tourists in a targeted manner and let them interactively experience the characteristic folk culture and the representative intangible cultural heritage of the destination, form recognition of the overall field of interactive perception, space atmosphere, environmental image perception, theme and characteristics, service attitudes and activities, and gradually form place attachment and place identity to traditional villages, so as to enhance tourists’ perception of place attachment, improve their well-being and loyalty to the destination, and ultimately affect their post-travel behavior.
3) Enhance the immersive experience of tourists in traditional village tourism through improving design styles, characteristic products, folk activities, service attitudes, memory recalling and experience perception, which can create immersive situational experience for tourists. Attention should also be paid to meeting the needs of tourists for self-relaxation and entertainment when setting up recreation perception interactions. In addition, the feelings at the level of emotional resonance can be published through social media, and personalized service experience can be spread through high-quality word-of-mouth after travel, so as to improve the marginal benefits and communication benefits of experiential marketing.

It is necessary to constantly pay attention to the influencing factors of tourists' revisit intention, timely adjust the tourism routes and items of tourist destinations to maintain the attractiveness of traditional villages, appropriately disclose the data and results of tourists' revisit intention, promote market potential through content marketing to attract more tourists who have never been there, cultivate and enhance their revisit intention during the tour, and finally form a closed consumption loop of the tourist destination.

In conclusion, it is necessary to explore and amplify the differentiated characteristics of the advantages of traditional villages and cultivating them into a regional culture carrier that integrates space atmosphere, place attachment, perceived interest, experiential marketing, recreation perception, environmental image perception and information richness can enhance the competitiveness of traditional villages and promote the sustainable development of traditional village tourism.

### 6.2 Research limitations and future possibilities

In this research, several limitations are presented. First of all, the subjects of questionnaire are mainly 18–35 years old, which ignores elder tourist groups most of whom have rural life experience, and their emotional connection with traditional villages forms their strong willingness to return to traditional villages. In addition, the married accounted for more than 2/3, the influence of many factors may be related to family behavior and family composition, which is not included in the discussion of this research. Therefore, researchers may pay more attention to this issue in the future. Secondly, due to COVID-19, the target population of this research is domestic tourists, and their feedback cannot represent the driving factors of traditional village tourism for international tourists. No sample data has been formed for international tourists who have traveled to traditional villages one or more times. It is suggested that researchers prepare multilingual questionnaires in the future to enrich sample types and cover other regions such as Asian countries, North America, Europe and the Middle East, so as to set up a wider range of participants and discuss the influencing factors of international tourists’ travel to traditional villages. Last but not least, this research focuses on the construction of model, lacking in in-depth and specific discussion on each single dimension, especially how to use smart technology to improve the well-being and tourists’ revisit intention to traditional villages with the rapid development of information and communication technology.

## Funding

This research was funded by University Research Base of Humanities and Social Sciences in Fujian Province- Project of Research Center of Cross-media Design (Grant No.16KPSS03). This research was also supported by Rural Culture Research Institute of Fujian Jiangxia University and 2021 Fujian Jiangxia University School level scientific research projects (Grant No.JXS2021010).

## Compliance with ethical standards

### Ethical approval

The study was conducted in accordance with the Declaration of Helsinki, and approved by the Institutional Review Board of Bohai University (protocol code 2021.983746, approval date 2021.4.21).

Verbal informed consent was obtained from the patients for their anonymized information to be published in this article.

## CRediT authorship contribution statement

**Mengyi Lin:** Writing – original draft, Visualization, Validation, Software, Methodology, Formal analysis, Data curation, Conceptualization.

## Declaration of competing interest

The authors declare that they have no known competing financial interests or personal relationships that could have appeared to influence the work reported in this paper.
