# Supplementary material for: Understanding the influencing factors of tourists’ revisit intention in traditional villages
Source: Heliyon. 2024 Jul 22;10(15):e35029. doi: 10.1016/j.heliyon.2024.e35029 (PMC11320434; doi:10.1016/j.heliyon.2024.e35029)
Supplement: Multimedia component 1 [file mmc1.docx]

**Survey questionnaire on consumer experience factors in traditional village tourism**

This is a survey questionnaire about consumer experience factors in traditional village tourism, aiming to understand your actual experience in the process of traditional village tourism. This questionnaire is anonymous and not intended for commercial purposes. It is only for academic research and there is no right or wrong answer. Please rest assured to answer. Thank you for your assistance.

Best Wishes

Mengyi Lin

**Basic information**

**1.Gender**

- Male
- Female

**2.Age**

- 18-25
- 26-35
- 36-45
- Over 46

**3.Education level**

- Junior high school and below
- Technical secondary school and high school
- College and undergraduate
- Master degree and above

**4.Profession**

- Student
- Civil servant and employee of public institutions
- Company employee
- Self-employed
- Professional technical staff skill
- Retired
- Other

**5.Marriage**

- Married
- Unmarried

**6.Average income per month**

- Below 3000RMB
- 3000-6000RMB
- 6000-10000RMB
- 10000-15000RMB
- Over 15000RMB

**The following are questions about your travel experience. Please choose the most suitable answer based on your recent experience traveling to a traditional village.**

1. Space Atmosphere ( Spatial environment and atmosphere presented by traditional villages, including architectural style, natural landscapes, cultural atmosphere, and other factors, which influence the perception and emotional experience of tourists. )

| **Item** | Strongly disagree | Disagree | Neutral | Agree | Strongly Agree |
| --- | --- | --- | --- | --- | --- |
| The architectural layout of this traditional village is reasonable. |  |  |  |  |  |
| The traditional village has a complete range of entertainment facilities. |  |  |  |  |  |
| The staff in this traditional village treat people with enthusiasm and attentive service. |  |  |  |  |  |
| The cultural architecture of this traditional village blends perfectly with the natural scenery. |  |  |  |  |  |

1. Place Attachment （ The emotional attachment and sense of identification that tourists have towards the area where the traditional village is located, including the emotional connection and degree of attachment to the local culture, history, and customs.）

| **Item** | Strongly disagree | Disagree | Neutral | Agree | Strongly Agree |
| --- | --- | --- | --- | --- | --- |
| I am most satisfied with the folk culture in this traditional village. |  |  |  |  |  |
| The traditional village gives me the feeling that other places cannot give it. |  |  |  |  |  |
| This traditional village is the best place for me to experience rural folk culture in a short time. |  |  |  |  |  |
| I very much agree with the rural folk culture displayed by this traditional village. |  |  |  |  |  |
| For me, this traditional village has a special meaning. |  |  |  |  |  |
| I admire the environment of this traditional village very much. |  |  |  |  |  |

1. Perceived interest playfulness （The perceived novelty and attractiveness of traditional village tourism activities and experiences, including the perceived novelty of local activities, cultural performances, handicrafts, and so on.）

| **Item** | Strongly disagree | Disagree | Neutral | Agree | Strongly Agree |
| --- | --- | --- | --- | --- | --- |
| For me, visiting this traditional village is very interesting. |  |  |  |  |  |
| For me, visiting this traditional village is very novelty. |  |  |  |  |  |
| During visiting this traditional village, it makes me feel happy to share my travel experience. |  |  |  |  |  |

1. Experiential Marketing （The marketing strategies and implementation effects of traditional village tourism products and services, including the perception and evaluation of aspects such as visitor experience design, service quality, and promotional efforts.）

| **Item** | Strongly disagree | Disagree | Neutral | Agree | Strongly Agree |
| --- | --- | --- | --- | --- | --- |
| The design style of this traditional village is in line with my aesthetics. |  |  |  |  |  |
| The characteristic products displayed in this traditional village are attractive to me. |  |  |  |  |  |
| The folk activities in this traditional village are familiar to me. |  |  |  |  |  |
| Being in this traditional village can remind me of the past. |  |  |  |  |  |

1. Recreation Perception （ Perception and Experience of Tourists on the Leisure and Entertainment Value of Traditional Villages, including their perception and evaluation of scenic facilities, leisure activities, culinary experiences, and more.）

| **Item** | Strongly disagree | Disagree | Neutral | Agree | Strongly Agree |
| --- | --- | --- | --- | --- | --- |
| This traditional village can meet my needs for relaxation and entertainment. |  |  |  |  |  |
| During visiting this traditional village, I managed to adjust my emotions. |  |  |  |  |  |
| This traditional village can achieve my purpose of recreation. |  |  |  |  |  |

1. Environmental Image Perception （Tourists' overall image and impression of the natural environment and cultural landscape of traditional villages, including their perception and evaluation of cleanliness, aesthetics, cultural atmosphere, and more.）

| **Item** | Strongly disagree | Disagree | Neutral | Agree | Strongly Agree |
| --- | --- | --- | --- | --- | --- |
| The overall environment of this traditional village is comfortable and tidy. |  |  |  |  |  |
| The traditional village has fully equipped dining and entertainment facilities. |  |  |  |  |  |
| All the consumption and commodity prices in this traditional village are reasonable. |  |  |  |  |  |
| The traffic volume in this traditional village is suitable. |  |  |  |  |  |
| The traffic planning in this traditional village is reasonable and parking is convenient. |  |  |  |  |  |

1. Information Richness （The degree to which tourists obtain and perceive information about traditional village tourist destinations, including the richness of information about scenic spot introductions, historical culture, activity arrangements, and more.）

| **Item** | Strongly disagree | Disagree | Neutral | Agree | Strongly Agree |
| --- | --- | --- | --- | --- | --- |
| In addition to relaxing my mind, this traditional village also displayed ancient rural folk culture costumes and architecture. |  |  |  |  |  |
| In addition to relaxing my mind, this traditional village can also tell me a lot of knowledge about rural folk culture . |  |  |  |  |  |
| In addition to relaxing my mind, this traditional village also conveys correct traditional cultural values. |  |  |  |  |  |
| When traveling in this traditional village, I learned the meaning of many traditional cultural symbols. |  |  |  |  |  |

1. Tourism Well-being （The happiness and satisfaction generated by tourists during the process of visiting traditional villages, including the emotional experiences of pleasure, relaxation, and fulfillment brought about by the travel experience.）

| **Item** | Strongly disagree | Disagree | Neutral | Agree | Strongly Agree |
| --- | --- | --- | --- | --- | --- |
| Overall, I am very satisfied with this traditional village tour. |  |  |  |  |  |
| I think the journey is full of fun. |  |  |  |  |  |
| I have been very enthusiastic during this trip. |  |  |  |  |  |
| I feel joy and excitement during this journey. |  |  |  |  |  |

1. Revisit Intention （The willingness of tourists to choose traditional villages as a travel destination again, including their satisfaction with the travel experience and their expectations for future revisits.）

| **Item** | Strongly disagree | Disagree | Neutral | Agree | Strongly Agree |
| --- | --- | --- | --- | --- | --- |
| I am going to visit this traditional village again next time. |  |  |  |  |  |
| I may come to this traditional village again in the future. |  |  |  |  |  |
| This traditional village will be my first choice for experiencing rural folk culture in the future. |  |  |  |  |  |
| I will introduce relatives and friends to visit this traditional village. |  |  |  |  |  |
